# Rational Design of Artificial Metalloproteins and Metalloenzymes with Metal Clusters

**DOI:** 10.3390/molecules24152743

**Published:** 2019-07-29

**Authors:** Ying-Wu Lin

**Affiliations:** 1 School of Chemistry and Chemical Engineering, University of South China, Hengyang 421001, China; linlinying@hotmail.com or ywlin@usc.edu.cn; Tel.: +86-734-8578079; 2 Laboratory of Protein Structure and Function, University of South China, Hengyang 421001, China; 3 Hunan Key Laboratory for the Design and Application of Actinide Complexes, University of South China, Hengyang 421001, China

**Keywords:** metalloproteins, metalloenzymes, protein design, metalclusters, synthetic models

## Abstract

Metalloproteins and metalloenzymes play important roles in biological systems by using the limited metal ions, complexes, and clusters that are associated with the protein matrix. The design of artificial metalloproteins and metalloenzymes not only reveals the structure and function relationship of natural proteins, but also enables the synthesis of artificial proteins and enzymes with improved properties and functions. Acknowledging the progress in rational design from single to multiple active sites, this review focuses on recent achievements in the design of artificial metalloproteins and metalloenzymes with metal clusters, including zinc clusters, cadmium clusters, iron–sulfur clusters, and copper–sulfur clusters, as well as noble metal clusters and others. These metal clusters were designed in both native and de novo protein scaffolds for structural roles, electron transfer, or catalysis. Some synthetic metal clusters as functional models of native enzymes are also discussed. These achievements provide valuable insights for deep understanding of the natural proteins and enzymes, and practical clues for the further design of artificial enzymes with functions comparable or even beyond those of natural counterparts.

## 1. Introduction

Metalloproteins and metalloenzymes play important roles in biological systems, including electron transfer, O_2_ binding and delivery, and catalysis, etc. [1,2,3,4,5,6,7,8,9,10,11]. Despite the functional diversity, the cofactor or prosthetic group of native metalloproteins/metalloenzymes are made of about 14 metal ions, and several types of metal complexes (such as heme) or metal clusters (such as iron–sulfur clusters) [4,5,10], which may limit the functionalities of native metalloenzymes. To overcome these limitations, it is desirable to rationally design artificial metalloproteins and metalloenzymes, which have received great attention for several decades, and achieved significant progresses, such as for artificial oxidases and reductases, etc. [12,13,14,15,16,17,18,19,20,21,22,23,24,25,26,27,28,29]. These progresses not only reveal the key structural elements responsible for the activity of native enzymes, but also endows the ability to create artificial enzymes with improved properties and functions.

In the design of artificial metalloproteins and metalloenzymes, many strategies have been developed, such as redesign of the active metal site by fine-tuning the cofactor–protein interactions through site/loop-directed mutagenesis [30,31,32], the design of new metal-binding sites [33,34,35,36,37,38], the incorporation of unnatural amino acids and non-native cofactors into native or de novo protein scaffolds [20,25,39,40,41], and the directed evolution of metalloenzymes [23,42], as well as the use of post-translational modifications (PTMs) [43,44,45,46,47,48,49,50]. Other materials such as nanoparticles and hydrogels have also been used for the design of enzyme mimics [51,52,53]. Moreover, these designs have been achieved for metalloproteins and metalloenzymes with single to multiple active sites by construction of the mononuclear site via metal substitution or incorporation, the design of homodinuclear or heterodinuclear sites and the reconstitution of metal complexes, as well as the design of dual and multiple active sites in single, dimeric proteins, and protein oligomers and polymers [15]. The progress in this field has also been well reviewed very recently [21,22,23,24,25,26,27,28,29].

**Figure 1:**
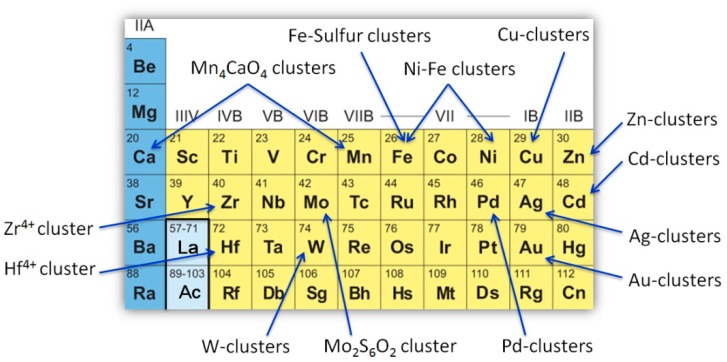
Artificial metalloproteins and metalloenzymes with diverse metal clusters shown in the periodic table.

Comparatively, there has been less progress in the design of artificial metalloproteins and metalloenzymes with metal clusters, especially for those formed by multiple metal ions. In 2016, Fehl and Davis critically reviewed the progress in the design of heterogeneous catalysis by using proteins or polypeptides as templates for synthetic metalloclusters, including iron–sulfur clusters, di-iron clusters, and nickel–iron clusters [54]. In 2018, Ueno et al. highlighted the research on the functionalization of protein crystals with metal ions, complexes, and nanoparticles [55]. In this review, we summarize the very recent progress in the rational design of artificial metalloproteins and metalloenzymes by focusing on the design of multi-metal clusters, as well as some synthetic metal clusters as functional models of native enzymes. The mapping of the diverse metal clusters that have been designed to date in the periodic table is shown in Figure 1. These achievements provide not only valuable insights into the structure–function relationship of native metalloproteins and metalloenzymes, but also practical clues for creating more advanced artificial metalloenzymes, which will ultimately stimulate the growth and expansion of this field.

## 2. Artificial Metalloproteins with Metal Clusters for Structural Roles

### 2.1. Zinc Clusters

**Figure 2:**
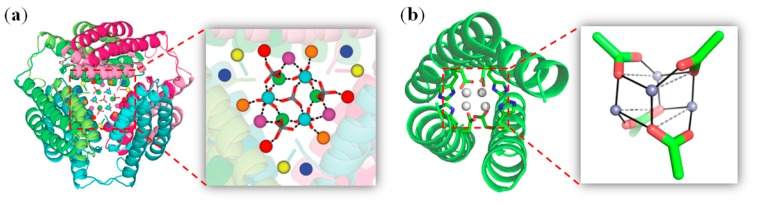
(**a**) Cage structure of three domain-swapped Cyt*cb*_562_ dimers (PDB code 5AWI), and a close-up view of the Zn^2+^ and SO_4_^2−^ ions in the internal cavity. Reprinted with permission from Ref. [59], Copyright 2016 The Royal Society of Chemistry; (**b**) Crystal structure of 4DH1 (PDB code 5WLL), and a close-up view of the designed tetranuclear zinc cluster. Reprinted with permission from Ref. [63], Copyright 2018 American Chemical Society.

Zinc ions play crucial roles in both protein scaffolds and protein–protein interfaces, acting as catalytic sites or supporting quaternary protein structures [56]. Tezcan et al. showed that by design of azinc-binding site on the protein surface, such as on *c*-type cytochrome *b*_562_ (Cyt*cb*_562_) with a covalently attached heme, the protein assembles in ordered structures as dictated by the binding of Zn^2+^ ions [57]. Both structural zinc sites (3-His-1-Asp) and catalytic zinc sites (2-His-1-Glu-1-H_2_O) can be designed in Cyt*cb*_562_ assembly, which confer a stable and active artificial hydrolase both *in vitro* and in vivo [58]. Note that Cyt*cb*_562_ is a four-helix bundle protein, and can form a dimer by domain-swapping, wherethe *N*-terminal two helices of one protomer may interact with the C-terminal two helices of the other protomer. Moreover, Hirota et al. observed that three domain-swapped Cyt*cb*_562_ dimers can further form a unique nanocage, witha novel Zn–SO_4_ cluster (15 Zn^2+^ and 7 SO_4_^2−^ ions) inside the cavity, as shown in the X-ray crystal structure (Figure 2a) [59]. In addition to the coordination between Zn^2+^ and SO_4_^2−^ ions, the Zn^2+^ ions in the Zn–SO_4_ cluster were also coordinated by the amino acid side chains of the dimers, which stabilized the cage structure. Additional stabilization effects were contributed from a hinge loop thatconnected two four-helix bundle units.

As an alternative to native proteins, de novodesigned proteins such as helical bundles provide ideal scaffolds for the design of artificial metalloproteins by the incorporation of metal ions, metal complexes, or metal clusters [36,60]. For example, Pecoraro et al. designed a Zn^2+^-binding site (3-His-1-H_2_O) inthree-helical bundles, which confers an impressive hydrolase activity toward the hydration of CO_2_ with an efficiency comparable to that of native carbonic anhydrases [61]. DeGrado et al. designed a dinuclear zinc site in four-helical bundles, with two Zn^2+^ ions bridged by two Glu residues and coordinated by additional His and Glu residues. With a suitable substrate-binding pocket, this de novo protein was able to stabilize the radical semiquinone form of catechols for weeks, which is otherwise unstable in aqueous solution [62]. Recently, Lombardi et al. designed a tetranuclear zinc cluster within four-helical bundles (named 4D/EH1/2), which consist of four Zn^2+^ ions and four carboxyl oxygens from Asp/Glu (Figure 2b) [63]. Additional ligands were provided by His residues, which were further stabilized by second-shell and third-shell interactions, forming a fully connected H-bond network. By optimizing the amino acid sequence, the peptide can be designed to form a tetramer in aqueous solution in the absence of metal ions, which subsequently binds Zn^2+^ ions and forms a tetranuclear cluster, suggesting an impact of the designed geometry to the metal cluster [64].

### 2.2. Cadmium Clusters

**Figure 3:**
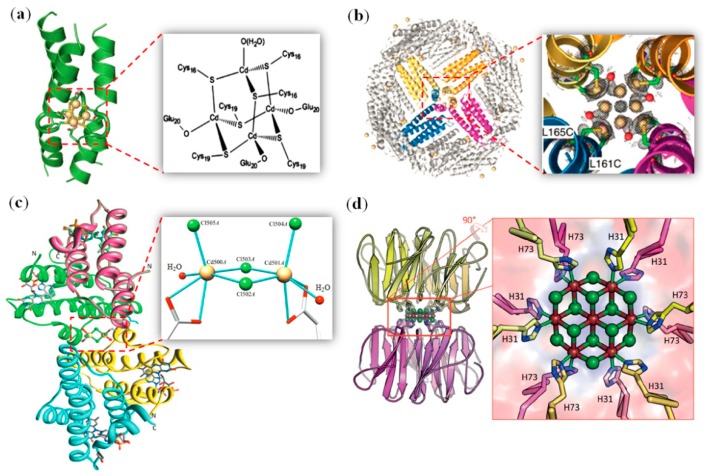
(**a**) X-ray crystal structure of a tetranuclear Cd–thiolate cluster (PDB code 4G1A), and the coordination structure. Reprinted with permission from Ref. [67], Copyright 2013 Elsevier; (**b**) X-ray crystal structure of apo-L161C/L165C-Fr with Cd ions bound (PDB code 6JEE), and a close-up view of the fourfold axis channel. Reprinted with permission from Ref. [70], Copyright 2019 The Royal Society of Chemistry; (**c**) X-ray crystal structure of tetrameric hsALR (PDB code 3R7C), and a close-up view of the Cd_2_Cl_4_O_6_ cluster. Reprinted with permission from Ref. [71]. Copyright 2012, International Union of Crystallography; (**d**) X-ray crystal structure of nvPizza2-S61H58 (PDB code 5CHB), and a close-up view of the Cd_7_Cl_12_ cluster. Reprinted with permission from Ref. [72], Copyright 2015 Wiley-VCH.

Similar to a tetranuclear copper cluster (Cu_4_S_4_) that can be designed within a four-helical bundle [65,66], a tetranuclear cadmium cluster can also be designed, such as in the interior of a three-helical bundle, by using a metal-binding motif of CXXCE [67]. As shown by X-ray crystallography (Figure 3a), the tetra-Cd^2+^ clusteris a tetrahedral adamantane-like cluster, with four Cd^2+^ ions bridged by six Cys residues and coordinated by three Glu residues, as well as an additional water molecule, i.e., [Cd_4_(μ_2_-S·Cys)_6_(O_2_C·Glu)_3_(H_2_O)], resulting in a very stable de novo designed metalloprotein.

Ferritin (Fr) is a cage-like protein for iron storage, which is formed by the assembly of 24 subunits [68]. As a result, the structure is highly symmetrical, with twofold, threefold, and fourfold symmetry axes. The threefold axis channel is hydrophilic, and can be used for metal penetration. Differently, the fourfold axis channel has a hydrophobic microenvironment. Inspired by the use of de novofour-helical bundles for the design of metal-binding sites [69], Ueno et al. introduced two Cys residues (L161C/L165C or L168C/L69C mutation) at the fourfold axis channel of apo-Fr, which leads to the formation of four or eight binding sites for Cd^3+^ ions, as revealed by the X-ray crystal structures [70]. For example, the X-ray structure of apo-L161C/L165C-Fr showed that a Cd_8_-cluster is located at the fourfold axis channel (Figure 3b), which is coordinated by Cys161 and/or Cys165 with a distance of 2.4 to 2.5 Å, as well as water molecules (2.4 Å). The Cd–Cd distance was found to be 3.4 Å, which agrees with thoseof the Cd–S cluster in natural metallothionein (3.4 to 4.4 Å), suggesting the possible formation of a metal–metal bond, although the occupancies of both Cd ions were less than one (0.6 and 0.2, respectively). This study suggests that not only the threefold axis channel, but also thefourfold axis channelof Fr can be used for design of metal clusters, with the coordination structure controlled by the assembly interfaces of protein cages.

In addition to the Cd–sulfur cluster, cadmium can form clusters with inorganic ions such as chloride ions. Rose et al. found that when crystallized, the protein augmenter of liver regeneration containing a 14-residue hexahistidine purification tag (termed hsALR) in the presence of a high concentration of CdCl_2_ (50 mM), the protein formed a tetramer composed of two homodimers, which was bridged by a novel Cd_2_Cl_4_O_6_ cluster with coordination by two Asp residues, as well as two water molecules (Figure 3c) [71]. Moreover, the formation of a cadmium chloride cluster can be rationally designed by usage of the protein interface. As designed by Voet et al., an artificial β-propeller protein named Pizza forms a trimer with threefold symmetry. By the further design of metal-binding sites using His as ligands, the new version of nvPizza2-S61H58 was found to accumulate cadmium and chloride ions by dimerization of the trimer, forming a novel Cd_7_Cl_12_ cluster at the threefold symmetric axis (Figure 3d) [72]. The cadmium ions were coordinated by the His residues from each subunit, as well as chloride ions, resulting in a lattice that is nearly identical to that of crystalline cadmium chloride. This study suggests that symmetric proteins may be used for the biomineralization of nanocrystals with useful properties.

### 2.3. Noble Metal Clusters

In general, noble metals refer to eightelements, including ruthenium (Ru), rhodium (Rh), palladium (Pd), silver (Ag), osmium (Os), iridium (Ir), platinum (Pt), and gold (Au). They tend to form metal clusters with a sub-nanometer size (<2 nm), which have a diverse array of applications in biology, energy, and the environment [73]. Moreover, the formation of noble metal clusters with desirable sizes can be controlled by rationally designed metal-binding proteins. For example, Morozov and Ogawa [74] designed helical bundles to form trimers, tetramers, and hexamers, which can bind six, eight, and 12 Ag^+^ ions, respectively. Upon chemical reduction, a series of Ag^0^ nanoclusters with predictable sizes can be formed, which display strong visible fluorescence with a number-dependent emission energy. Recently, Cortajarena et al. developed a simple strategy to design proteins for the sustainable synthesisof metal nanoclusters by the introduction of a dihistidine coordination site [75]. Using this approach, they obtained metal nanoclusters such as Cu cluster (15 ± 1 atoms), Ag cluster (nine ± 1 atoms), and Au cluster (five ± 1 atoms) within the protein scaffold of a 34-amino acid helix–turn–helix motif, which showed photostable luminescence both in vitro and in vivo, thereby allowing biomedical applications such as cell imaging and labeling.

**Figure 4:**
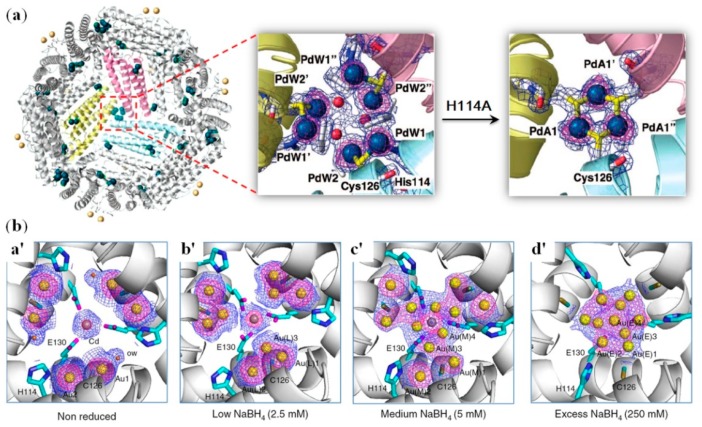
(**a**) Pd(allyl) complexes binding to the threefold channel of apo-Fr, which form a trinuclear Pd-cluster as a result of H114A mutation. Reprinted with permission from Ref. [76], Copyright 2008 American Chemical Society. (**b**) X-ray crystal structures Au-loaded apo-Frs in the absence and presence of different concentrations of NaBH_4_, showing the process of formation of an Au-cluster at the threefold channel of apo-Fr [77].

As shown in the previous section, Fr is a highly symmetrical cage-like protein. In its threefold axis channel, the presence of both His and Cys residues allows the coordination of noble metal ions, such as Pd^2+^ and Au^3+^ ions. These metal ions can also be designed to form metal clusters. Ueno et al. showed that metal complexes of Pd^II^(allyl) (allyl = η^3^-C_3_H_5_) can be accumulated in the threefold axis channel of apo-Fr, forming a thiol-bridged dinuclear structure, which was stabilized by the coordination of His114 and a water molecule (Figure 4a, middle) [76]. When His114 was replaced with a non-coordinating Ala residue, a unique thiolato trinuclear Pd-cluster was formed, with a six-membered ring structure (Figure 4a, right). This observation suggests that the threefold axis channel of apo-Fr can be modified to acquire metal ions and form novel metal clusters.

Moreover, as shown in Figure 3b, Ueno et al. observed the formation of a gold sub-nanocluster at the threefold axis channel of apo-Fr [77]. By the introduction of two surface Cys residues, the apo-E45C/R52C-Fr protein with Au^3+^ ions loaded were first cross-linked together to form stable crystals, which showed the binding of multiple Au^3+^ ions at the threefold axis channel with His114, Cys126, and H_2_O as the ligands, as well as a Cd^2+^ ion, due to the use of (NH_4_)_2_SO_4_/CdSO_4_ as the precipitant for crystallization (Figure 4ba′). Upon the reduction of these Au^3+^ ions by using different concentrations of NaBH_4_, the X-ray structural analysis showed the gradual movement of the Au^3+^ ions and the ultimate formation of a sub-nano gold cluster (totally 10 Au^3+^ ions), which accompanied significant conformational changes of the His114 residue (Figure 4(bb′–bd′)). Therefore, these observations deepened our understanding of metal cluster formation as well as its interactions with the protein microenvironments.

### 2.4. Other Metal Clusters

**Figure 5:**
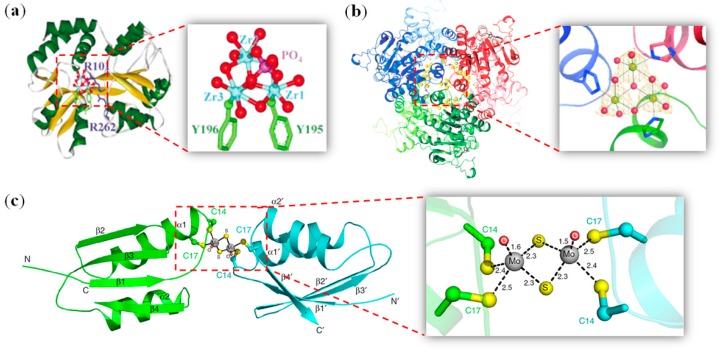
(**a**) X-ray structure of Zr_3_–nFbp showing the coordination geometry. Reprinted with permission from Ref. [79]. Copyright 2004 Wiley–VCH; (**b**) Structure of the Mo/WSto protein of *A. vinelandii*, and a close-up view of the oxo-W_3_-cluster. Reprinted with permission from Ref. [80], Copyright 2007 Wiley-VCH; (**c**) X-ray structure of a dimer of the metal-binding domain ofcellular copper efflux protein ATP7B (WLN4), and a close-up view of a cluster Mo_2_S_6_O_2_ at the interface [81].

In addition to those metal clusters mentioned in previous sections, other non-nativemetal clusters can also be formed in the protein scaffolds with a structural role, which were either rationally designed or found in experiments. For example, Sadler et al. showed that an ferric–ion-binding protein from *Neisseria gonorrhoeae* (nFbp) may form trinuclear or pentanuclear oxo-clusters of Hf^4+^ and Zr^4+^ using a di-tyrosyl cluster nucleation motif (Tyr195-Tyr196) [78,79]. An X-ray crystal structure of Zr_3_–nFbp was shown in Figure 5a, which revealed an oxo-Zr_3_ phosphate cluster that was coordinated directly by both Tyr195 and Tyr196 to two of the three Zr^IV^ ions, whereas the O atoms of the cluster were H-bonded to surrounding amino acids [79]. Müller et al.observed several types of polynucleartungsten oxide clusters (W_2_, W_3_, W_6_, W_7_, and W_7+x_) in the binding cavity of a Mo/W-storage protein, and a close-up view of the oxo-W_3_-cluster was shown in Figure 5b [80].

Recently, Liu et al. found that ammoniumtetrathiomolybdate ([(NH_4_)_2_MoS_4_], TM) can induce the dimerization of the metal-binding domain ofcellular copper efflux protein ATP7B (WLN4), due to the formation of a unique sulfur-bridged Mo_2_S_6_O_2_ at the interface, as shown by the X-ray crystal structure in Figure 5c [81].The detailed structure showed that the Mo atom formstwo Mo=O double bonds, as indicated by the distances between Mo and O (1.5 and 1.6 Å), which is in a pyramidalgeometry, with coordination from six S atoms (four Cys and two inorganic S atoms) at a distance of 2.3 to 2.5 Å.

## 3. Artificial Metalloproteins/Metalloenzymes with Metal Clusters for ElectronTransfer

Nature has evolved diverse electron-transfer centers for metalloproteins, which includes single metal ions such as iron and copper, metal complexes such as *b*-type and *c*-type hemes, and metal clusters such as different iron–sulfur clusters ([2Fe-2S], [3Fe-4S] or [4Fe-4S]), with a broad range of reduction potentialsfrom −700mV to +800 mV [7]. Due to their important rolesof electron transfer in biology, as well as serving as catalysts, iron–sulfur clusters are of considerable interest in the design of artificial metalloproteins and metalloenzymes, which aimed at gaining insights into the complex nature systems, and possible applications in industry and energy [82,83]. With a deep understanding of structural principles for natural iron–sulfur proteins, the design of artificial iron–sulfur proteins hasobtained significant progress [83].

**Figure 6:**
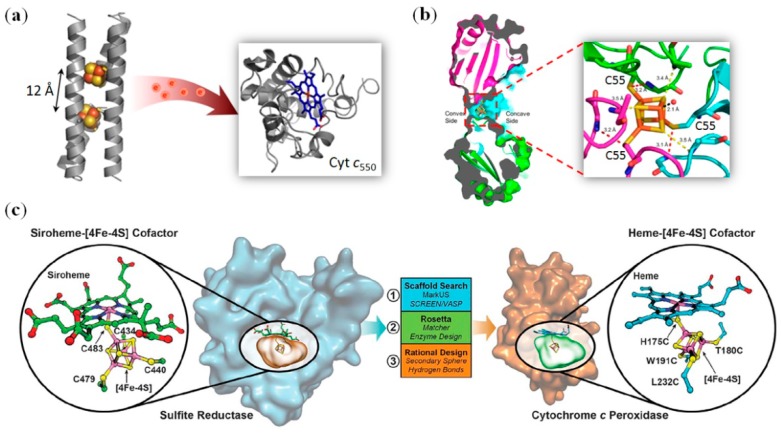
(**a**) Rational design of two [4Fe-4S] clusters in domain-swapped dimer(DSD)-Fdm that transfer electrons to ferric Cyt *c*. Reprinted with permission from [86]. Copyright 2014 American Chemical Society; (**b**)X-ray structure of BMC-T1-S55C and a close-up view of the [4Fe-4S] cluster (PDB code 5DII). Reprinted with permission from [89]. Copyright 2014 American Chemical Society; (**c**) Rational design of a [4Fe-4S] cluster in cytochrome *c* peroxidase (C*c*P) that closely mimics the active site of native sulfite reductase (SiR). Reprinted with permission from [98]. Copyright 2018 AAAS.

In a pioneer work, with the help of computer-aided design, Goldren et al. introduced four Cys residues into the hydrophobic core of thioredoxin, followed by the incorporation of a [4Fe-4S] cluster into the protein scaffold [84]. Similarly, by the introduction of eight Cys residues in the hydrophobic core of a dimeric three-helix bundle (domain-swapped dimer, DSD), Ghirlanda et al. that two cubane-type [4Fe-4S] clusters was readily bound by in situ reconstitution, which increases the thermal stability relative to that of apo-protein. Meanwhile, the two clusters were separated by 29–34 Å, and were not efficientin electron transfer [85]. In a second generation of protein design, these authors shortened the distance between two clusters to 12 Å (Figure 6a), which is a distance comparable to native intercluster distance (≤15 Å), such as in ferredoxin [86]. In addition to structural mimicking, the designed protein, named DSD-Fdm, showed a reduction potential (–479 mV, [4Fe-4S]^2+/1+^) within the lower range of natural ferredoxins. As a result, the reduced protein was able to transfer electrons to ferric Cyt*c*_550_ (Figure 6a), with a stoichiometry of Cyt*c*_550_/DSD-Fdm = 2/1, which closely mimics the function of natural ferredoxins. Note that the protein reduction potential could be fine-tuned by ligand replacement. For example, the replacement of one of the Cys ligands with Ser or Leu increased thereduction potentialto −4 mV and 12 mV, respectively [87]. More recently, Falkowskia et al. designed artificial symmetric ferredoxins that bind two [4Fe-4S] clusters and exhibit reduction potentials ranging from −405 mV to −515 mV, which are capable of shuttling electrons in vivo, such as through designed cellular pathways in *E. coli* [88].

Similar to other metal clusters mentioned in previous sections, the iron–sulfur cluster can also be designed within the protein interface if it forms a suitable binding pocket. For example, Kerfeld et al. redesigned the interface of a trimeric bacterial microcompartment (BMC) shell protein by introducing a single Cys at position 55, namely BMC-T1-S55C [89]. The designed protein was found to spontaneously incorporate a [4Fe-4S] cluster at the threefold symmetry axisof the trimer when the protein was expressed in vivo. As shown by X-ray structure (Figure 6b), the cluster was coordinated by three Cys55 residues, as well as an additional water molecule, which provides the first structural information for a designed [4Fe-4S] cluster within a protein scaffold. Moreover, the cluster exhibited a reduction potential of −370 mV (pH 7.5); this value is close to that of the minimal ferredoxin maquette (−350 mV, pH 8) [90], whereas it is more positive than that of dimeric ferredoxin maquette (−479 mV, pH 7.5) [86]. This comparison suggests that greaterexposure of the [4Fe-4S] cluster to the solution leads to more positive potential, which in turn suggests that the reduction potential of the [4Fe-4S] cluster can be fine-tuned by modifying the environment hydrophobicity.

Metalloenzymes with multi-cofactors are efficient in catalyzing multi-electron reactions, such as heme-copper oxidases (HCO, Cu_B_-heme dinuclear center) [91,92,93], nitric oxide reductases (NOR, Fe_B_-heme dinuclear center) [34,94,95,96], and sulfite reductase (SiR, heme-[4Fe-4S] center) [48,97,98]. It is full of challenges to reproduce both the structure and function of these native enzymes by the design of artificial enzymes with a heteronuclear cofactor. Lu et al. have made significant achievements in this field. In addition to the design of both the structural and functional model of HCO and NOR in the scaffold of Mb, namely Cu_B_Mb [92] and Fe_B_Mb 34], respectively, they recently designed an artificial SiR in the scaffold of C*c*O (Figure 6c) [98]. With the help of the Rosetta matcher, four residues were selected and mutated to Cys (H75C, T180C, W191C, and L232C) to coordinate to the iron of [4Fe-4S], where Cys175 acts as an axial ligand the heme iron. As shown by the modeling structure in Figure 6c, the designed enzyme closely mimics the active site of native SiR. To further optimize the microenvironment of the heme active site to improve substrate binding and catalysis, triple mutations (W51K, H52R, and P145K) were performed, which enhances the reductase activity by ~5.3-fold. Moreover, an H-bond was further introduced to the [4Fe-4S] cluster by D235N or D235C mutation, resulting in a 17-fold and 63-fold increase over that of the first generation, which indicates that both the microenvironment and H-bond interactions are key factors affecting the catalytic efficiency. Note that the highest rate of sulfite reduction achieved was 21.8 min^−1^, which is ~18% that of a native SiR (121 min^−1^). This study demonstrates that the rational design of a [4Fe-4S] cluster in combination with secondary interactions may confer upon artificial enzymesan ability to performmulti-electron and multi-proton reduction.

## 4. Artificial Metalloenzymes with Metal Clusters for Catalysis

### 4.1. Iron–Sulfur Clusters

**Figure 7:**
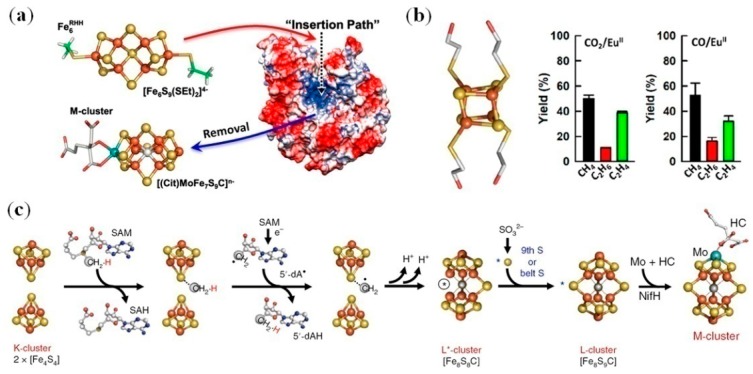
(**a**) An artificial metalloenzyme constructed by replacement of the M-cluster with a synthesized [Fe_6_S_9_(SEt)_2_]^4−^ cluster in nitrogenase scaffold. Reprinted with permission from [102]. Copyright 2015 Wiley-VCH; (**b**) The structure of [4Fe-4S(SCH_2_CH_2_OH)_4_]^2−^ cluster (left), and its catalysis of CO_2_/CO reduction in the presence of europium(II) diethylenetriaminepentaacetic acid (Eu^II^-DTPA) (right) [104]. Copyright 2018 Nature press; (**c**) The proposed mechanism of l-cluster assembly in nitrogenase, followed by the maturation of M-cluster. SAM: S-adenosyl-l-methionine; NifH: the reductase component of Mo-nitrogenase;HC:homocitrate. Reprinted with permission from [103]. Copyright 2018 Macmillan Publishers.

As mentioned in the previous section, iron–sulfur clusters play rolesof both electron transfer and catalysis in biology. For example, nitrogenase catalyzes the reduction of nitrogento ammonia, as well as other substrates (C_2_H_2_, CN^−^, etc.), at its cofactor center of [(Cit)MoFe_7_S_9_C]^n−^ (designated M-cluster), where electrons were transferred from a [4Fe–4S] cluster via an [8Fe–7S] cluster (designated P-cluster) [99]. The M-cluster consists of [Mo–3Fe–3S] and [4Fe–3S] subclusters, as bridged by three S atoms and one carbide (C^4−^) atom (Figure 7a) [100]. To mimic this unique geometry and the anionic nature, Holm et al. synthesized a hexanulcear, dithiolateiron–sulfur cluster, [Fe_6_S_9_(SEt)_2_]^4−^, which was designated Fe_6_^RHH^ (Figure 7) [101]. To reveal whether the M-cluster mimic could be combined with a protein scaffold to afford a functional enzyme, Hu et al. removed the native M-cluster from the catalytic component of nitrogenase, followed by incorporation of the Fe_6_^RHH^ cluster into the apo-protein (Figure 7a) [102]. The reconstituted artificial enzyme was able to catalyze the reduction of C_2_H_2_ to C_2_H_4_ in the presence of the reductase component of nitrogenase and ATP, with an activity comparable to that of the native enzyme. Moreover, in the absence of ATP and presence of a strong reductant, europium(II) diethylenetriaminepentaacetic acid (Eu^II^-DTPA), the artificial enzyme can catalyze the reduction of CN^−^ to C_1_-C_3_ hydrocarbons, with CH_4_ being the major product. This study demonstrates that the functional artificial enzyme can be obtained by the combination of a synthetic cluster with an appropriate protein scaffold.

Recently, thesemi-synthetic approach was extended to reveal the assembly mechanism of the M-cluster. For determination of the ninth sulfur source of the l-cluster [Fe_8_S_9_C], Hu et al. synthesized a water-stable and soluble [4Fe-4S] cluster with four –SCH_2_CH_2_OH ligands (designated [4Fe-4S]^syn^, Figure 7b, left) that were exchangeable by Cys residues from protein, and incorporated it into the apo-protein of nitrogenase [103]. The results showed that SO_3_^2−^, instead of S^2−^ or SO_4_^2−^, gives rise to the ninth sulfur, with the formation of a new [Fe_8_S_8_C] cluster intermediate, namely the L*-cluster. Then, the M-cluster was maturated by the insertion of Mo and homocitrate (HC) (Figure 7c). Moreover, the cluster of [4Fe-4S]^syn^ was found to be able to catalyze the reduction of C1 substrates such as CO_2_ and CO in the presence of Eu^II^-DTPA, producing hydrocarbons (CH_4_, C_2_H_4_, and C_2_H_6_) in an aqueous buffer (Figure 7b, right) [104,105]. These observations indicate an inherent catalytic feature for the [4Fe-4S] cluster, which may thus be used for the design of other artificial metalloenzymes.

### 4.2. Copper–Sulfur Clusters

**Figure 8:**
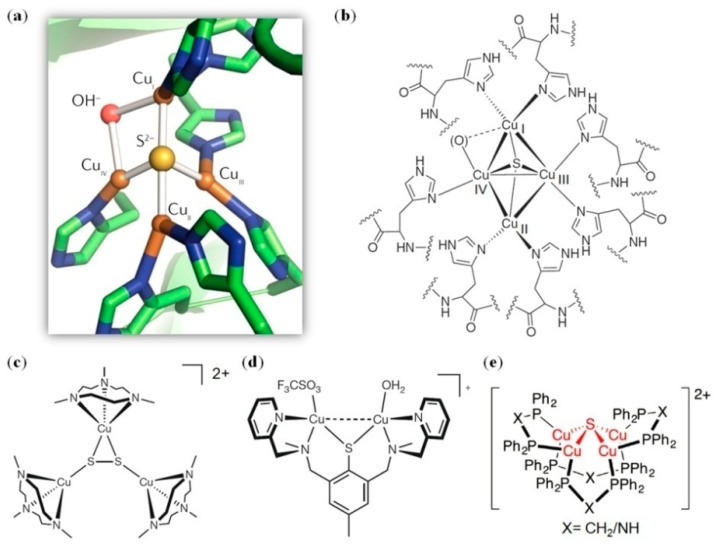
(**a**) The X-ray structure of the Cuz center in N_2_OR from *P. nautica* (PDB code 1QNI [112]); (**b**) The coordination geometry of the Cuz center in native N_2_OR; (**c**–**e**) The structures of the synthetic functional models of the native Cuz center [117,118,119].

Copper is an essential element in biological systems. When associated with a protein matrix by coordination with His, Cys, and/or Met, it plays diverse functions including O_2_ binding, electron transfer, and catalysis [106,107]. Copper centers have various types, such as the Cu_A_ and Cu_B_ centers found in cytochrome *c* oxidase (C*c*O) that contain two and one copper atom, performing electron-transfer and catalysis functions, respectively [106,107]. It was revealed that the axial Met ligand and the surrounding amino acids influence the reduction potential of Cu_A_ via coordination, H-bond, and hydrophobic interactions [107,108,109], and in the active site of the Cu_B_ center, a novel Tyr–His cross-link was found to fine-tune the protein reactivity [110,111]. In addition, the Cu_Z_ center was discovered in N_2_O reductase (N_2_OR), which containsfour copper atoms forming a distorted tetrahedral geometry, with an inorganic sulfur ion (S^2-^) as a bridging ligand (Cu_4_S cluster, Figure 8a, PDB code 1QNI [112]). The copper ions (Cu_I-IV_) were coordinated by seven His residues, and the Cu_I_–Cu_IV_ edge was bridged by one unknown O ligand (Figure 8a,b), which was proposed to be the active site where the substrate binds and reduces to N_2_ and H_2_O (N_2_O + 2e^−^ + 2H^+^ → N_2_ + H_2_O) [113,114].

It is full of challenges to design an artificial enzyme with a Cuz center, and no report is availablein the literature yet. Meanwhile, some progresses have been made in the design of synthetic model complexes mimicking the structure and function of the Cuz cluster in native N_2_OR [115,116]. For example, Cramer et al. reported the first functional model of Cuz with a [Cu_3_(μ_3_-S_2_)] core (Figure 8c), which exhibited spectroscopic features similar to those of N_2_OR, and can reduce N_2_O to N_2_ under mild conditions such as at room temperature [117]. Instead of using a bridging S^2−^ ligand, Esmieu et al. synthesized a dissymmetric mixed-valentdicopper(II,I) complex using thiolates, which contains a [Cu_2_S] core with labile triflate and water molecules at the copper centers (Figure 8d) [118]. This simple dicopper complex was shown to be a functional model of the Cuz cluster, by the reduction of N_2_O to N_2_at room temperature. Recently, Mankad et al. reported a tetranuclear copper cluster with a [Cu_4_(μ_4_-S)] core (Figure 8e), in its one-hole (S=1/2) redox state [119]. The one-hole clusterwas shown to reduce N_2_O and produce N_2_, which is the first model closely mimicking the one-electron reduced form of native Cuz (termed Cuz*, Cu^I^_4_S). Therefore, although these model complexes work in organic solvents such as CH_2_Cl_2_ and acetone, they are very useful for gaining the structure and function relationship of native N_2_OR. Moreover, the incorporation of these model complexes in suitable protein scaffolds might be able to produce artificial metalloenzymes working in physiological conditions.

### 4.3. Other Metal Clusters

**Figure 9:**
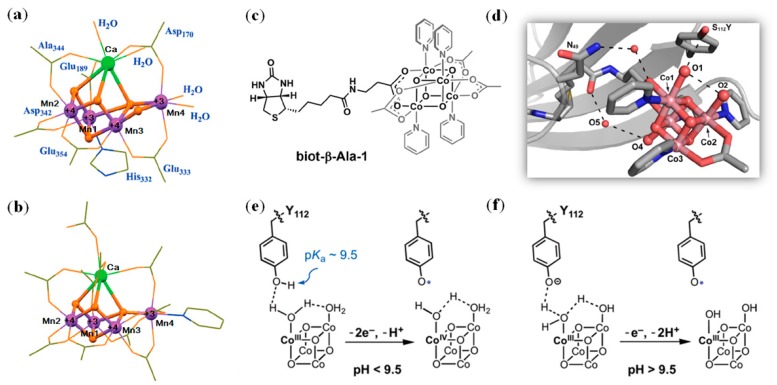
(**a**) The X-ray crystal structure of native oxygen-evolving center (OEC) showing the core Mn_4_CaO_5_ cluster and the ligands; (**b**) The X-ray crystal structure of a synthesized Mn_4_CaO_4_ cluster; Reprinted with permission from [121], Copyright 2015 AAAS; (**c**) The chemical structure of biotinylated Co_4_O_4_ cluster, biot-β-Ala-1; (**d**) The X-ray crystal structure of 2xm-S112Y-Sav with biot-β-Ala-1 bound (PDB code 6AUE); (**e**–**f**) Proposed mechanisms for the multi-e^−^/multi-H^+^ reactivity of the designed enzyme at pH <9.5 or pH >9.5.Reprinted with permission from [123]. Copyright 2018 American Chemical Society.

The oxygen-evolving center (OEC) in photosystem II has received much attention in the last decade, which contains a Mn_4_CaO_5_ cluster for water oxidation (Figure 9a). Many effects have been directed to synthetic mimics of the OEC. For example, Agapie et al. have rationally synthesized a [Mn_3_CaO_4_]^6+^ cubane that structurally models the Mn_3_Ca subsite of OEC [120]. Zhang et al. have synthesized a Mn_4_CaO_4_ cluster closely mimicking the native OEC, not only in the metal–oxygen core, but also in the coordinating amino acids with ligands of acetate and pyridine (Figure 9b) [121]. Note that the two water ligands on the Ca^2+^ ion could be mimicked not only by acetate, but also by polar solvents such as acetonitrile and DMF, as structurally characterized recently by Zhang et al. [122]. Moreover, the artificial Mn_4_CaO_4_ cluster underwent four redox transitions similar to that of native OEC, which thus provides an ideal model for studying the structure and function relationship of native OEC, as well as an artificial enzyme model for applications in water oxidation.

Since the above-synthesized cluster was not incorporated into a protein scaffold, the secondary coordination sphere interactions could hardly be fine-tuned. Meanwhile, in a native photosystem II system, Tyr residue facilitates protein-coupled electron transfer (PCET) between the OEC center and light-harvesting chlorophylls (P680). It is challengeable to design an artificial metalloenzyme with tunable PCET properties. Recently, Tilley et al. synthesized a biotinylated Co_4_O_4_ cluster (Figure 9c) and incorporated it into a Sav protein by employing the biotin-streptavidin technology [123]. By introduction of a proximal Tyr (S112Y mutation), the X-ray crystal structure revealed that Tyr112 forms an H-bond interaction with the water molecule that acts as an axial ligand of the Co ion (Figure 9d). As a result, a chemistry of multi-e^−^/multi-H^+^ was observed in electronchemical studies, with a transition of the mechanism at pH 9.5 corresponding to the pKa value (~9.5) of Tyr (Figure 9e,f). Note that in the absence of the proximal Tyr (Ser112 or Phe112), only 1e^−^/1H^+^ chemistry was observed, which suggests that the secondary sphere interactions, as provided by the redox active Tyr residue, are critical for fine-tuning the multi-e^−^/multi-H^+^ reactivity for the artificial enzyme [123]. Note that the introduction ofa redox active residue such as Tyr and Trp in the heme distal pocketor on the protein surface was also shown to fine-tune the reactivity of artificial peroxidases designed in the protein scaffold of myoglobin [46,124,125].

## 5. Conclusions and Perspectives

In summary, artificial metalloproteins and metalloenzymes with diverse metalclusters have been rationally designed in recent years, in which the clustersare formed either in the protein pocket, between the interface of protein dimers, trimers, and oligomers, or within the scaffold of de novo designed proteins. As shown in Figure 1, the metal ions used for the design of metalclusters are not only those in natural biological systems, such as the first-row transition metals (Mn, Co, Fe, Cu, and Zn, Figure 2 and Figure 6, Figure 7, Figure 8 and Figure 9), but also non-native metal ions, such as those of the second-row transition metals (Zr, Mo, Pd, Ag, and Cd, Figure 3, Figure 4a and Figure 5a,c) and the third-row transition metals (Hf, W, and Au, Figure 4b and Figure 5b). Although only several amino acids (Cys/Met, His, and Asp/Glu) act as the ligand for the metal clusters, the bridging atoms/ligands are diverse, which are usually inorganic anions such as S^2−^, SO_4_^2−^, Cl^−^, O^2−^, and OH^−^, and amino acids (Cys/Asp/Glu) (Figure 2, Figure 3, Figure 4, Figure 5, Figure 6, Figure 7, Figure 8 and Figure 9), as well as synthetic ligands such as –SCH_2_CH_2_OH (Figure 7b). In most cases, these metal clusters were found to play a structural role, which stabilize the protein 3D structures, dimers, trimers, or oligomers. Moreover, the metal clusters, especially iron–sulfur clusters, have been successfully designed to play roles of both electron-transfer and catalysis (Figure 6 and Figure 7). Within this progress, some functional model complexes have been rationally designed and synthesized, which closely mimic the active metal clusters in more complex native metalloenzymes, such as the Cuz center in N_2_OR (Figure 8b–e) and Mn_4_CaO_5_ cluster in photosystem II (Figure 9a,b), respectively.

Currently, the design of artificial metalloproteins and metalloenzymes with metal clusters is still at the stage of providing insights into the structure and functional relationship for native metalloenzymes, and some synthetic models are still waiting for incorporation into suitable protein scaffolds to work in physiological conditions. Comparatively, artificial metalloenzymes with a single active site have been designed to exhibit catalytic parameters similar to those of native enzymes [50,98,126,127], or even with a much higher catalytic efficiency [128], which have potential applications in the future. Moreover, some artificial metalloenzymes may catalyze reactions beyond the functionalities of natural enzymes, such as the catalytic formation of a C–Si bond by an engineered Cyt *c* [129]. By the construction of multi-metal clusters in protein scaffolds, the catalysis of multi-electron/multi-proton reactions has been achieved [98,123], which otherwise can hardly be achieved by using a single metal center, suggesting a more promising application in biotransformations. As suggested by Martinez et al., oxidoreductases are on their way from laboratory to industry [130]. We are confident that in the near future, more advanced artificial metalloenzymes with metal clusters will be rationally designed andexplored for practical applications in different fields, such as in biological medicine, biofuel generation, and environmental protection.
